# High output cardiac failure in 3 patients with hereditary hemorrhagic telangiectasia and hepatic vascular malformations, evaluation of treatment

**DOI:** 10.1186/s13023-020-01583-6

**Published:** 2020-11-26

**Authors:** Lilian B. Olsen, Anette D. Kjeldsen, Mikael K. Poulsen, Jens Kjeldsen, Annette D. Fialla

**Affiliations:** 1 HHT-Center Odense University Hospital, Part of VASCERN, Odense, Denmark; 2 Department of Otorhinolaryngology Head and Neck Surgery, Odense, Denmark; 3 Department of Cardiology, Odense University Hospital, Odense, Denmark; 4 Department of Medical Gastroenterology and Hepatology, Odense University Hospital, Odense, Denmark; 5 Institute of Clinical Research, Odense, Denmark

**Keywords:** Hereditary hemorrhagic telangiectasia, HHT, Vascular endothelial growth factor inhibitor, Bevacizumab, Hepatic, Right heart failure

## Abstract

**Background:**

This report addresses how patients with hereditary hemorrhagic telangiectasia (HHT) and high output cardiac failure (HOCF) due to hepatic vascular malformations, should be evaluated and could be treated. HHT is a genetic disorder, leading to vascular abnormalities with potentially serious clinical implications. In the liver, arteriovenous malformations occur in more than 70% of patients, but only about 8% present clinical symptoms such as HOCF with pulmonary hypertension and less commonly portal hypertension, biliary ischemia and hepatic encephalopathy.

**Results:**

Three female patients with HHT type 2 and HOCF caused by severe arteriovenous malformations in the liver are presented in this case series. The patients were seen at the HHT-Centre at Odense University Hospital. Treatment with either orthotopic liver transplantation (one patient) or bevacizumab (two patients) was initiated. All patients experienced marked symptom relief and objective improvement. New York Heart Association—class were improved, ascites, peripheral edema and hence diuretic treatment was markedly reduced or discontinued in all three patients. Bevacizumab also resulted in notable effects on epistaxis and anemia.

**Conclusion:**

Our findings substantiate the importance of identification of symptomatic arteriovenous malformations in the liver in patients with HHT. Bevacizumab may possibly, as suggested in this case series and supported by previous case studies, postpone the time to orthotopic liver transplantation or even make it unnecessary. Bevacizumab represents a promising new treatment option, which should be investigated further in clinical trials.

## Introduction

Hereditary hemorrhagic telangiectasia (HHT) is a genetic disorder, leading to vascular abnormalities with potentially serious clinical implications [1, 2]. It is an autosomal dominant disease with an estimated prevalence of 1:6.500 in Denmark [3]. It is commonly associated with multiple telangiectatic lesions at characteristic sites as the skin of the face and fingertips. HHT also manifests with telangiectatic lesions of the mucosa causing troublesome bleedings and potentially anemia. Finally, the patients may have visceral arteriovenous malformations (AVMs) in particular in the lungs, liver and central nervous system which can result in severe organ dysfunction and failure [4–7].

The HHT-diagnosis is established by the Curaçao criteria, which include spontaneous, recurrent epistaxis, telangiectasia in HHT-associated locations, visceral involvement and a family history [8].

The mutations involved in HHT most commonly affect genes coding for the Endoglin (ENG) and Activin receptor-like kinase 1 (ACVRL1) causing HHT type 1 and type 2, respectively [9, 10]. In rare cases, HHT is caused by mutations of the SMAD4 gene leading to a phenotype with concomitant juvenile polyposis [10, 11]. The common denominator of these mutations is that they result in loss of function of receptors belonging to the transforming growth factor beta (TGFβ) superfamily, which are present in endothelial cells, involved in angiogenesis and considered to be the cause of vascular dysfunction in HHT [12, 13].

The most common symptom in HHT is epistaxis occurring in more than 90% of patients [14, 15]. Another common clinical presentation is dyspnea and cyanosis due to AVMs in the lungs [16–18].This is caused by shunting of blood from the pulmonary arterial bed to the pulmonary veins, bypassing the lung tissue.

Furthermore, telangiectatic lesions in the gastrointestinal (GI)-tract cause bleeding in up to 25% of patients and consequently cause anemia [19].

In the liver, AVMs occur in more than 70% of patients with HHT, but only about 8% present with clinical symptoms [4]. The AVMs form between the hepatic arteries and the liver veins causing high output cardiac failure (HOCF) with pulmonary hypertension, or between the hepatic arteries/or veins and the portal veins, causing portal hypertension, biliary ischemia and hepatic encephalopathy [20]. HOCF is characterized by high cardiac output secondary to an increased blood volume. In HHT-patients increased blood flow through liver AVMs, eventally lead to heart-failure. The patients may be diagnosed with HOCF while the underlying cause (the liver-AVMs) may be overlooked.

Treatment options for HHT with HOCF due to liver AVMs progressing to terminal HOCF have until now been limited. Thus, in terminal HOCF, orthotopic liver transplantation (OLT) has been the only treatment option [21–23].

Bevacizumab is a recombinant monoclonal antibody, which inhibits vascular endothelial growth factor (VEGF), a signal protein, which, among other functions, stimulates angiogenesis. It is commonly used in antineoplastic treatment [24–26].

In case reports and minor, non-randomized, studies on HHT patients with symptomatic liver AVMs, pharmacological treatment with bevacizumab has been shown to markedly reduce cardiac output and simultaneously reduce frequency and duration of epistaxis and GI-bleeding [27–31].

In this case series, three cases with HOCF caused by severe liver AVMs, treated with either OLT or bevacizumab are presented.

## Methods

Three female patients two with confirmed and one with possible (VUS) HHT type 2 (Table 1), all treated in 2018 at the HHT-Centre at Odense University Hospital (OUH), are presented. The clinical evaluations and treatments were performed in collaboration between the Departments of Oto-Rhino-Laryngology, Cardiology and Medical Gastroenterology and Hepatology.

**Table 1:** Baseline characteristics

	Patient 1^a^	Patient 2^a^	Patient 3
Age (years)	65	80	66
Sex	Female	Female	Female
HHT-type	2	2	VUS^d^
Gene mutation	ACVLR-1-c.1022A>T	ACVLR-1-c.1022A>T	ACVLR-1-c.941A>C^b^
Height (cm)	161	163	160
Weight (kg)	53	60	47
Body Mass Index	21	23	18
PAVM	Yes^c^	No	No
GI-AVM	Yes	Yes	No
Epistaxis	Yes, mild	Yes, mild	Yes, severe

^a^Patients are related

^b^Not formerly known to be pathogenic

^c^5 cm in diameter, embolized in 2009

^d^Having a variant of unknown significance in the ACVRL1 gene, means that we found a mutation but we do not know if it is a disease causing mutation or just a polymorphic variant. The conclusion for this patient is that the patient has HHT based on the clinical Curaçao criteria, but we cannot say for sure she has HHT type two

*ACVLR-1* activin receptor-like kinase 1, *VUS* variance of unknown significance, *PAVM* pulmonary arteriovenous malformation, *GI-AVM* gastrointestinal-AVM

One patient was referred for OLT and two patients were treated with bevacizumab. Bevacizumab was administered during 6 series at a three-week interval, by intravenous infusion at a dose of 5 mg per kilogram of body weight followed by maintenance infusion every 3 months. The follow-up period for all three patients was 9 months.

A standard clinical evaluation is performed on patients referred to the national HHT-Centre under the suspicion of HHT. The work-up includes patient history, focusing on the Curaçao criteria as well as neurological symptoms, dyspnea and other cardiac symptoms. Blood samples are collected for blood count and genetic testing is performed to assess the disease causing mutation and if possible establish the disease subtype.

An index echocardiogram is performed in all HHT-patients at our center, regardless of symptoms. Contrast echocardiography is used as the first screening tool for pulmonary AVMs [32–35]. If this indicates pulmonary AVMs, a computed tomography (CT)-angiography is performed to identify AVMs. Secondly, if symptoms later on indicate congestive heart disease an echocardiogram is performed, at the time of symptoms. If the echocardiogram shows signs of pulmonary hypertension and/or right heart failure (right ventricular dilatation and/or reduced systolic function) a right heart catheterization is performed. Since congestive heart disease is a potential manifestation of hepatic AVMs, such findings always yield a follow-up CT angiography of the abdomen.

### Ethics

Written informed consent was obtained from all patients, allowing their disease history to be published. The data are enclosed in the Danish HHT database, Danish Data Protection Agency (Jnr13/42,552).

## Results

Patient 1 was a 65-year-old woman with HOCF symptoms and a Cardiac Index (CI) of 6.6 L/min/m^2^, caused by liver AVMs (Tables 1, 5). During her first visit at the outpatient clinic, her symptoms were dyspnea, New York Heart Association (NYHA) class II, peripheral edema and atrial fibrillation (AF). She did not receive anticoagulation.She experienced epistaxis less than once a month (Tables 1, 2). An abdominal CT-scan revealed hepatomegaly, dilatation of the hepatic artery and vein and a magnetic resonance cholangiopancreatography showed signs of cholangiopathy, suggestive of hepatobiliary ischemia (Table 3). Furthermore, she had previously had a pulmonary AVM, which had successfully been treated with endovascular coil-embolization in 2009 (Table 1).

**Table 2:** Cardiac measures

	Patient 1		Patient 2		Patient 3	
	Before	After OLT	Before	After BVZ	Before	After BVZ
NYHA-class	II	I	II	I	III	I
AF	Yes^a^	Yes^a^	No	No	Yes	Yes
Peripheral edema	Yes	No	Yes	Reduced	Yes	Reduced

^a^Paroxysmal

*OLT* orthotopic liver transplantation, *BVZ* bevacizumab, *NYHA* New York Heart Association, *AF* atrial fibrillation

**Table 3:** Liver measures

	Patient 1		Patient 2		Patient 3	
	Before	After OLT	Before	After BVZ	Before	After BVZ
Ascites	No	No	No	No	Yes	No
Hepatic artery^a^ (mm)	12.6	–	11.5	11.5	11.7	9.5
Superior Mesenteric artery^b^ (mm)	6.5	–	6.5	6.5	9.5	6
Portal vein^c^ (mm)	8.5	–	17	22	23.5	19
Hepatic vein^d^	22.3	–	15	15	16	14
Hepatobiliary ischemia	Yes	No	No	No	No	No
Diuretic treatment	Yes	No	Yes	Reduced	Yes	Reduced
Transfusion	No	No	Yes	No	No	No

^a^Measured 10–20 mm from coeliac truncus

^b^Measured 5 mm downstream from the branching off from the aorta

^c^Measured at the crossing of the hepatic artery

^d^Measured 5 mm from the inferior vena cava

*OLT* orthotopic liver transplantation, *BVZ* bevacizumab

Patient 1 underwent OLT in July 2018 at the referral transplant centre in Copenhagen. Following transplantation her CI immediately decreased from 6.6 to 4.5 L/min/m^2^. She suffered from post-surgical bleeding from a phrenic artery, inducing a drop in hemoglobin from 10.5 g/dL to 8.1 g/dL within 24 h. An abdominal ultrasound revealed an organized 1.3 L hematoma, posterior to the liver, which was drained by a small surgical procedure. Subjectively, dyspnea was reduced, which improved her NYHA-class from II to I, allowing for discontinuation of her diuretic treatment, as peripheral edemas were significantly reduced (Tables 2, 3, 5).

At 9 month follow-up, patient 1 was feeling better than before OLT, although she had experienced a long period of hospitalization due to cholangitis, secondary to surgical complications, which was treated with drainage. She suffered from anemia without signs of bleeding. The immunosuppressant treatment (mycophenolate mofetil and tacrolimus) was still well tolerated. She did not have any side effects from the immunosuppressant treatment.

Patient 2 was an 80-year-old woman with liver AVMs causing HOCF, with a CI of 5.4 L/min/m^2^ (Tables 1, 5). She suffered from anemia caused by recurrent bleeding from gastrointestinal AVMs, dyspnea, NYHA class II and peripheral edema. Intermittently, she had required blood transfusions due to anemia (Tables 1, 2, 3, 4). OLT was not considered an option, due to very severe disease, advanced age and HOCF. The patient subsequently received bevacizumab treatment.

**Table 4:** Biochemistry before and after treatment

	Patient 1			Patient 2			Patient 3		
	Before	After OLT	9-months	Before	After BVZ	9-months	Before	After BVZ	9-months
Hemoglobin (g/L)	127.3	104.7	101.5	72.5	114.4	104.7	98.3	161.4	136.9
ALAT (U/L)	18	121	44	18	23	21	44	38	45
ALP (U/L)	89	215	232	112	117	100	125	85	100
Bilirubin (mg/dL)	0.58	1.05	0.7	0.88	–	–	1.4	1.58	25
INR	1.2	1.0	–	1.4	–	–	1.1	1.5	1.2
Bevacizumab side effects	No bevacizumab			Vulnerable and flossy nails			Dry, itchy skin, alopecia		

*OLT* orthotopic liver transplantation, *BVZ* bevacizumab, *ALAT* alanine aminotransferase, *ALP* alkaline phosphatase, *INR* international normalized ratio

After 18 weeks treatment, CI was reduced from 5.4 to 4.4 L/min/m^2^ (Table 5). NYHA-class had improved from II to I and diuretic treatment was reduced due to alleviation of peripheral edemas. Furthermore, during bevacizumab treatment gastrointestinal bleedings were reduced and the need for blood transfusions ceased (Tables 2, 3). She still did not experience epistaxis (Table 1). At 9 months follow-up she was feeling well and did not experience noteworthy side effects from bevacizumab, compared to the remarkable symptom relief she experienced. The only side effects reported by Patient 2 were vulnerable and flossy nails (Table 4).

**Table 5:** Cardiac data—before and after treatment

	Patient 1		Patient 2		Patient 3	
	Baseline	After OLT	Baseline	After BVZ	Baseline	After BVZ
ECG						
Rhythm	AFLI	AFLI	SR	SR	AFLI	AFLI
Heart rate (bpm)	77	90	95	83	86	74
Echocardiogram						
LV size	Normal	Normal	Normal	Normal	Normal	Normal
LVEF (%)	60	50	60	60	60	60
Left heart valve disease	No	No	No	No	No	No
RV size	Normal	Normal	Slight dilatation	Slight dilatation	Moderate dilatation	Moderate dilatation
TAPSE (mm)	34	14	31	24	17	20
TR size	Mild	Mild	Moderate	Moderate	Moderate	Moderate
TRG (mmHg)	43	30	36	41	65	39
RHC						
PCWP (mmHg)	18	NA	18	21	22	14
PAP, systolic (mmHg)	43	NA	52	60	78	51
PAP, diastolic (mmHg)	23	NA	21	21	32	22
PAP, mean (mmHg)	32	NA	38	40	48	32
RVP, systolic (mmHg)	42	NA	48	60	78	51
RVP, diastolic (mmHg)	10	NA	11	15	20	9
RAP (mmHg)	13	NA	12	12	18	9
Cardiac output (l/min)	10.2	6.5^a^	8.5	6.2	7.8	6.4
Cardiac index (l/min/m^2^)	6.6	4.5^a^	5.4	4.4	5.3	4.3
SVO_2_ (%)	90	NA	86	81	77	85
PVR (WU)	1.4	NA	2.4	3.1	3.7	2.8

^a^Perioperative

*OLT* orthotopic liver transplantation, *BVZ* bevacizumab, *AFLI* atrial fibrillation, *SR* sinus rhythm, *bpm* beats per minute, *LV* left ventricular, *LVEF* left ventricular ejection fraction, *RV* right ventricular, *TAPSE* tricuspid annular plane systolic excursion, *TR* tricuspid valve regurgitation, *TRG* max tricuspid regurgitation gradient, *RHC* right heart catheterization, *PCWP* pulmonary wedge pressure, *NA* not available, *PAP* pulmonary arterial pressure, *RVP* right ventricular pressure, *RA* right atrium pressure, *SVO2* pulmonic arterial oxygen saturation, *PVR* pulmonary vascular resistance, *WU* wood units

Patient 3 was a 66-year-old woman who was initially suspected to have liver cirrhosis. Following referral to the HHT-Centre, she was diagnosed with HHT and found to have liver AVMs and HOCF, with a CI of 5.3 L/min/m^2^ and symptoms of dyspnea, NYHA class III and AF, fatigue, anemia, ascites and peripheral edemas. She had heavy and prolonged epistaxis, also during the night, which was thought to be the cause of her anemia. Despite anemia she was treated with DOAC. The diagnosis of liver cirrhosis was rejected (Tables 1, 2, 3, 4). OLT was not considered an option due to very severe HOCF and pulmonary hypertension. She therefore received bevacizumab treatment.

Following 18 weeks of treatment, CI was reduced from 5.3 to 4.3 L/min/m^2^, NYHA-class improved from III to I. Her ascites had disappeared while her peripheral edemas alleviated and, hence, diuretic treatment was markedly reduced. Hemoglobin levels normalized (Tables 2, 3, 4, 5) and she no longer experienced epistaxis (Table 1). At 9 months follow-up she was feeling well. Patient 3 reported side effects in the form of itchy, dry skin and alopecia areata—a condition she had also suffered from before bevacizumab treatment (Table 4). The bevacizumab-related symptoms were considered insignificant by the patient compared to the remarkable symptom-relief she experienced.

## Discussion

In the current case series of three patients, OLT and treatment with bevacizumab both effectively reduced symptoms caused by liver AVMs. OLT in HHT patients with liver AVMs has previously been the treatment of choice in patients with severe symptoms despite serious adverse effects and complications [36–41]. However, limitations may be the age of the patient, comorbidity or severe right heart failure due to longstanding disease. If patients are excluded from OLT or waiting for OLT, bevacizumab treatment may be considered.

After OLT the liver AVMs are considered cured. However, the patients require life-long immunosuppressive treatment to reduce the risk of organ rejection [42]. One-year mortality following all-cause OLT is between 15–20% and post-transplantation life expectancy is significantly lower than in age-matched controls [43–45]. Additionally, OLT does not reduce the risk of bleeding from the gastrointestinal tract or the nasal mucosa, in contrast to findings during treatment with bevacizumab treatment [31].

Short-term effects of bevacizumab in HHT patients with liver AVMs, as presented in this case series have previously shown very promising results [29–31]. When focusing on the advantages of bevacizumab over OLT, treatment with bevacizumab exerts significantly less physical trauma to the patient as it is administered as an intravenous treatment with a low risk of inducing potentially fatal adverse effects [31, 46].

Thus, bevacizumab may be a significant and clinically relevant treatment option for patients with HHT with liver AVMs, although the liver AVMs are not cured with bevacizumab treatment, and long-term treatment might be necessary. Thus, bevacizumab may be a supplementary treatment option in patients before or in order to postpone or avoid OLT. Timing of OLT in HHT-patients with with liver AVMs and organ dysfunction is crucial. Pulmonary hypertension is a relative contraindication for OLT, and listing must be considered prior to this complication. When that is said, it is difficult to know whether there is a reversibility of both right heart failure and pulmonary hypertension, which leaves it difficult to decide, when the window closes for transplantation. Finally, due to the limited data available from bevacizumab treatment of non-cancer diseases, and specifically HHT, very little is known about adverse effects during long-term treatment. Continuous VEGF-inhibition could markedly affect normal physiological processes such as wound healing, vascular homeostasis and cardiovascular function [24, 47–51]. Furthermore, positive short-term effects, even immensely promising ones, are no guarantee of positive long-term effects.

## Conclusion

Identification of symptomatic liver AVMs in patients with HHT is important as they may result in significant clinical symptoms. Until now, OLT has been the mainstay in treatment and OLT leads to satisfying short- and long-term results in patients with HHT and liver AVMs. However, OLT potentially entails serious and in the worst-case life-threatening complications and adverse effects. Yet, treatment options for liver AVMs in patients with severe HHT are currently very limited, and there is no data on long-term effect. Thus, bevacizumab could, as suggested in this case series be a treatment option for patients with symptoms of HOCF. In some cases this may postpone or could even avoid the need to perform OLT. Bevacizumab represents a promising new treatment option, which should be investigated further in randomized clinical trials.

## Data Availability

All data are available upon contact to the corresponding author.

## Abbreviations

ACVR1: Activin receptor-like kinase 1
AF: Atrial fibrilation
AVM: Arteriovenous malformations
CI: Cardiac index
CT: Computed tomography
DOAC: Direct-acting oral anticoagulants
ENG: Endoglin
GI: Gastrointestinal
HHT: Hereditary hemorrhagic telangiectasia
HOCF: High output cardiac failure
OLT: Orthotopic liver transplantation
TGFβ: Transforming growth factor beta
NYHA: New York Heart Association
VEGF: Vascular endothelial growth factor inhibitor
